# Are Dietary Lectins Relevant Allergens in Plant Food Allergy?

**DOI:** 10.3390/foods9121724

**Published:** 2020-11-24

**Authors:** Annick Barre, Els J.M. Van Damme, Mathias Simplicien, Hervé Benoist, Pierre Rougé

**Affiliations:** 1 UMR 152 PharmaDev, Institut de Recherche et Développement, Université Paul Sabatier, Faculté de Pharmacie, 35 Chemin des Maraîchers, 31062 Toulouse, France; annick.barre@univ-tlse3.fr (A.B.); simplicien.mathias@gmail.com (M.S.); herve.benoist@ird.fr (H.B.); 2 Department of Biotechnology, Faculty of Bioscience Engineering, Ghent University, Coupure links 653, B-9000 Ghent, Belgium; ElsJM.VanDamme@UGent.be

**Keywords:** lectin, agglutinin, Ara h agglutinin, legume lectins, soybean lectin, garlic lectin, phytohemagglutinin, chitinase, allergen, IgE-binding epitope, allergenicity, food allergy

## Abstract

Lectins or carbohydrate-binding proteins are widely distributed in seeds and vegetative parts of edible plant species. A few lectins from different fruits and vegetables have been identified as potential food allergens, including wheat agglutinin, hevein (Hev b 6.02) from the rubber tree and chitinases containing a hevein domain from different fruits and vegetables. However, other well-known lectins from legumes have been demonstrated to behave as potential food allergens taking into account their ability to specifically bind IgE from allergic patients, trigger the degranulation of sensitized basophils, and to elicit interleukin secretion in sensitized people. These allergens include members from the different families of higher plant lectins, including legume lectins, type II ribosome-inactivating proteins (RIP-II), wheat germ agglutinin (WGA), jacalin-related lectins, GNA (*Galanthus nivalis* agglutinin)-like lectins, and Nictaba-related lectins. Most of these potentially active lectin allergens belong to the group of seed storage proteins (legume lectins), pathogenesis-related protein family PR-3 comprising hevein and class I, II, IV, V, VI, and VII chitinases containing a hevein domain, and type II ribosome-inactivating proteins containing a ricin B-chain domain (RIP-II). In the present review, we present an exhaustive survey of both the structural organization and structural features responsible for the allergenic potency of lectins, with special reference to lectins from dietary plant species/tissues consumed in Western countries.

## 1. Introduction

Besides their beneficial effects on human health, the consumption of plant food products may cause some detrimental effects, mainly associated with the presence of toxic molecules and allergenic proteins, which often occur in various plant parts, including leaves, roots, bulbs, fruits, and seeds [1,2]. Furthermore, the consumption of either crude or cooked foods from a variety of edible plant species is likely to trigger more or less severe allergic manifestations in both previously sensitized children and adults [3]. During the two last decades, huge progress has been achieved in terms of the identification and characterization of plant allergens responsible for these food allergies [4,5,6]. However, a lot of research remains to be done to obtain an exhaustive picture of the plant allergen diversity. In this respect, most of the relevant plant allergens consist of seed storage proteins, e.g., vicilins (7S globulin), legumins (11S globulin) and 2S albumins [7], and different families of pathogenesis-related (PR) proteins, e.g., PR-3 endochitinases [8], PR-5 thaumatin-like proteins (TLP) [9], PR-10 Bet v 1-like proteins [10], PR-12 defensins [11], and PR-14 lipid transfer proteins (LTP) [12], which all represent abundant proteins in seeds and other vegetative parts of edible plants. Among the protein allergens occurring in seeds and other vegetative tissues, the widely distributed plant lectins have been poorly investigated. Consequently, their role as relevant food allergens still remains a matter of debate [13]. Nevertheless, different families of lectins with various carbohydrate-binding specificities, have been identified and characterized in detail in all living organisms. Many of these lectins are rather abundant proteins in higher plants [14,15,16] (Table 1). A possible explanation for the lack of data on the allergenic potency of plant lectins relates to their ability to non-specifically bind to the carbohydrate moiety of IgE antibodies which most probably hampered the research in this area. However, this shortcoming can be overcome easily by performing the IgE-binding experiments in the presence of a cocktail of inhibiting sugars, e.g., mannose + galactose, that prevent the non-specific binding of plant lectins to the carbohydrate moiety of IgE from sera of allergic patients [17].

At present, a restricted number of higher plant lectins have been recognized unambiguously as potential allergens by the International Union of Immunological Societies (WHO/IUIS) [27]. In addition, many other plant lectins have been scarcely investigated with respect to their allergenic potential. Here, we present an exhaustive review of studies that have been devoted to the allergenicity of edible plant lectins. Furthermore, we propose some ideas for future research.

## 2. Allergenic Potential of Lectins from Higher Plants

In addition to their well-known mitogenic properties, essentially on T-lymphocytes, plant lectins are capable of inducing some allergic responses in previously sensitized people, similar to genuine allergens from plant foods and food products.

The list of lectins that have been assigned as potential food allergens by the WHO/IUIS Allergen Nomenclature Sub-Committee [27], comprises a restricted number of proteins including Tri a 18 (wheat germ agglutinin WGA) and a few chitinases with a hevein domain from banana (Mus a 2), turnip (Bra r 2), chestnut (Cas s 5), corn (Zea m 8), and avocado (Per a 1) (Table 1). Two other contact allergens, Hev b 6 (hevein) and Hev b 11 (class I chitinase containing a hevein domain) from the rubber tree *Hevea brasiliensis*, have been included in the list because they cross-react with food chitinases of classes I, II, IV, V, VI, and VII, which also possess a hevein domain.

In addition to this official list of lectin allergens, other plant lectins have been identified as potential food allergens following their ability to bind IgE, degranulate mast cells and basophils, and trigger the interleukin responses in various allergic patients [28,29,30]. These lectins have been included in Table 1 as non-IUIS approved potential food allergens (Table 2).

The non-IUIS approved lectin allergens consist of members from the different families of plant lectins, namely legume lectins, type II ribosome-inactivating proteins (RIP-II), wheat germ agglutinin, hevein and chitinases with a hevein domain, jacalin-related lectins, GNA-like lectins, and Nictaba-related lectins. All of these lectins exhibit very different structural scaffolds but share the common property to specifically recognize both simple sugars, e.g., mannose or galactose, and complex glycans, e.g., glycans of the *N*-acetyllactosaminic type or the high-mannose type [49].

### 2.1. Legume Lectins

Depending on the occurrence of a single or two polypeptide chains in their constitutive protomer and their quaternary association as homotetramers or homodimers, legume lectins are classified into two groups of closely-related lectins:

- the homotetrameric single-chain lectins, comprise both man-specific (e.g., Con A from Jackbean, *Canavalia ensiformis*, and DgL from *Dioclea grandiflora*) and Gal/GalNAc-specific lectins (e.g., PNA from peanut, *Arachis hypogaea*, and SBA from soybean, *Glycine max*).
- the homodimeric two-chain lectins, exclusively comprise man-specific lectins (e.g., LcA from lentil, *Lens culinaris*, and PsA from garden pea, *Pisum sativum*).

Both types of lectins have been identified as potential IgE-binding allergens.

#### 2.1.1. Homotetrameric Single-Chain Lectins

The Gal/GalNAc-specific lectin PNA from peanut (*Arachis hypogaea*), was among the first identified lectin allergens [43]. PNA is present in the protein bodies of the cotyledonary cells similar to other major allergens belonging to the group of seed storage proteins such as Ara h 1 (7S globulin/vicilin), Ara h 2 and Ara h 6 (2S albumin), and Ara h 3 (11S globulin/legumin). The lectin is designated as Ara h agglutinin and is considered a minor peanut allergen [43,50,51], At present, PNA is not officially recognized as a peanut allergen by the WHO/IUIS Committee.

**Figure 1:**
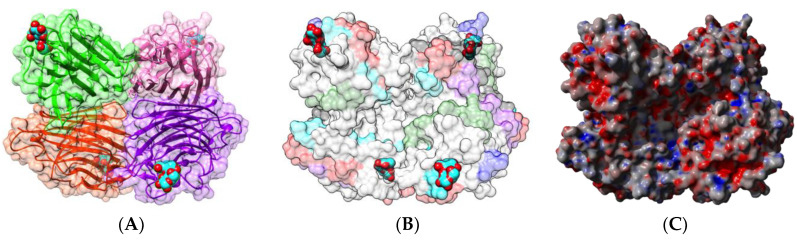
(**A**). Three-dimensional structure of PNA, showing the tetrameric arrangement of the lectin monomers (PDB code 2PEL). Secondary structure features are represented and the molecular surface is shown in transparency. Monomers I, II, III, and IV are colored green, pink, orange, and purple, respectively. Lactose molecules complexed to the carbohydrate-binding sites in each monomer are colored cyan. (**B**). Localization of the sequential IgE-binding epitopes identified at the molecular surface of the PNA homotetramer. Epitopes 1, 2, 3, 4, 5, and 6 are colored red (1), blue (2), green (3), grey (4), purple (5), and cyan (6), respectively. (**C**). Electrostatic potentials calculated at the molecular surface of PNA. (**A**,**B**) are not centered and thus, are truncated.

Native PNA occurs as a homotetramer built from the symmetrical non-covalent association of four identical protomers of 25 kDa, exhibiting the canonical jelly roll fold of legume lectins consisting of a β-sandwich made of two antiparallel β-sheets of 6 (front face) and 7 β-strands (back face), respectively (Figure 1A). Each protomer possesses a carbohydrate-binding site (CBS), which specifically recognizes Gal and GalNAc termini from unsialylated *N*-acetyllactosaminic glycans and the T/Tn-antigen expressed at the surface of many cancer cells [52].

Sequential IgE-binding B-cell epitopes identified on the surface of PNA [17] (Figure 1B), exhibit some amino acid sequence identity to other IgE-binding epitopes occurring at the surface of other legume lectins, such as LcA from lentil, PsA from pea, and SBA from soybean [53]. Most of the regions recognized as potential IgE-binding epitopes display both electropositively (colored blue) and electronegatively (colored red) charged residues, as shown from the calculation of surface electrostatic potentials at the molecular surface of PNA (Figure 1C). The homology between IgE-binding epitopes accounts for the cross-reactivity observed among legume lectins regarding their ability to interact with sera from peanut-allergic patients. However, this IgE-binding ability does not depend on the possible interaction of legume lectins with the carbohydrate moiety of IgE, since all the binding experiments were conducted in the presence of mannose and galactose, which specifically inhibit the lectin activity of both Gal/GalNAc-specific (PNA, SBA) and man-specific (LcA, PsA) legume lectins [53].

**Figure 2:**
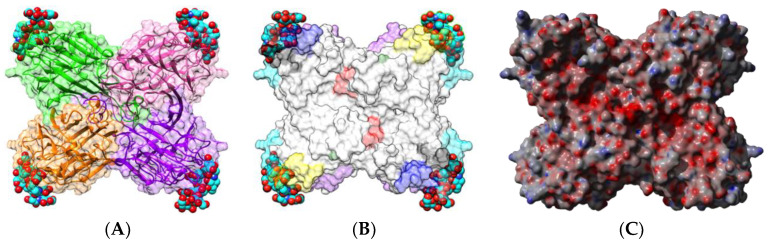
(**A**). Three-dimensional structure of the modeled PHA-E from black turtle bean (*Phaseolus vulgaris*), showing the tetrameric arrangement of the lectin monomers. Secondary structure features are represented and the molecular surface is shown in transparency. Monomers I, II, III, and IV are colored green, pink, orange, and purple, respectively. Bisected glycan chains complexed to the carbohydrate-binding sites in each monomer are colored cyan. (**B**). Localization of the sequential IgE-binding epitopes identified at the molecular surface of the PHA-E homotetramer. Epitopes 1, 2, 3, 4, 5, 6, and 7 are colored red (1), blue (2), green (3), grey (4), purple (5) yellow (6), and cyan (7), respectively. (**C**). Electrostatic potentials calculated at the surface of PHA-E. (**A**,**B**) are not centered and thus are truncated.

Kidney bean (*Phaseolus vulgaris*) seeds contain a mixture of erythroagglutinating (PHA-E) and leucoagglutinating (PHA-L) lectins, which interact with complex glycans of the *N*-acetyllactosaminic type, but do not recognize simple monosaccharides [53]. Both lectins consist of an arrangement different from that observed in PNA, containing four identical protomers possessing the canonical β-sandwich structure in a homotetrameric configuration (Figure 2A). Each protomer contains a CBS that specifically recognizes complex *N*-glycan chains, but does not recognize simple sugars, probably due to a particular conformation of the CBS.

**Figure 3:**
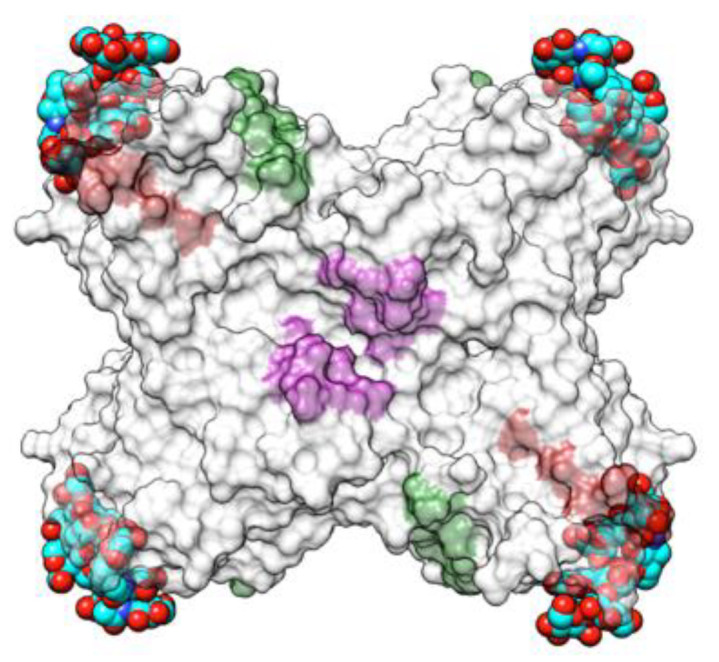
Three-dimensional structure of the modeled PHA-E from black turtle bean (*Phaseolus vulgaris*), showing the localization of T-cell epitopes 1, 2, and 3 identified by an in silico predictive approach. T-cell epitopes 1, 2, and 3 are colored green (1), red-brown (2), and magenta (3), respectively.

Both types of lectins from the white kidney bean [29,54,55,56,57,58] and the black turtle bean [59,60] have been reported as allergens, and a detailed bioinformatics study of the B- and T-cell epitopes of PHA-E from black turtle bean has been performed [59]. Up to 7 IgE-binding B-epitopes (Figure 2B) and 3 T-epitopes (Figure 3) occur at the surface of the PHA-E tetramer. Some overlap was identified between B-epitope #4 and T-epitope #2. Surprisingly, no B-epitopes were identified along the C-terminal region of the PHA-E amino acid sequence despite the pronounced sequence homologies between this region and the corresponding B-epitope-containing region of other legume lectins (see Figure 8 for explanation). Similar to PNA, some of the IgE-binding epitopic regions of PHA-E coincide with the presence of charged residues (Figure 2C).

**Figure 4:**
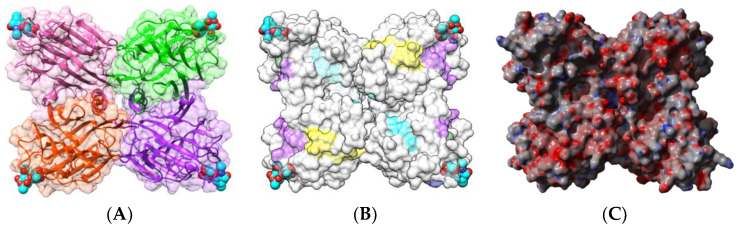
(**A**). Three-dimensional structure of the horse gram lectin DbA (PDB code 1LU1), showing the tetrameric arrangement of the lectin monomers. Secondary structure features are represented and the molecular surface is shown in transparency. Monomers I, II, III, and IV are colored pink, green, purple, and orange, respectively. Forssman disaccharides complexed to the carbohydrate-binding sites in each monomer are colored cyan. (**B**). Localization at the molecular surface of DbA of epitopic regions homologous to the IgE-binding epitopes identified in LcA and PsA. Homologous epitopic regions are colored red (1), blue (2), green (3), purple (4), yellow (5), and cyan (6), respectively. (**C**). Electrostatic potentials calculated at the surface of DbA. (**A**,**B**) are not centered and thus, are truncated.

**Figure 5:**
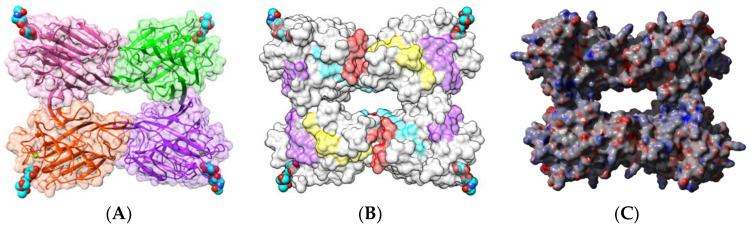
(**A**). Three-dimensional structure of soybean lectin SBA (PDB code 2SBA), showing the tetrameric arrangement of the lectin monomers. Secondary structure features are represented and the molecular surface is shown in transparency. Monomers I, II, II, I and IV are colored pink, green, purple, and orange, respectively. Disaccharides complexed to the carbohydrate-binding sites in each monomer are colored cyan. (**B**). Localization at the surface of the SBA tetramer of the IgE-binding epitopic regions homologous to the IgE-binding epitopes identified in LcA and PsA. Homologous epitopic regions are colored red (1), blue (2), green (3), purple (4), yellow (5), and cyan (6), respectively. (**C**). Electrostatic potentials calculated at the surface of SBA. (**A**,**B**) not centered and thus, are truncated.

Two single-chain Gal/GalNAc-specific lectins, DBA from horse gram (*Dolichos biflorus*) [44] and SBA from soybean (*Glycine max*) [45,61], have been identified as potential food allergens. They consist of homotetramers displaying a protomer arrangement very similar to that found in the kidney bean lectins PHA-E and PHA-L (Figure 4A and Figure 5A). Although no information is available on the occurrence of sequential IgE-binding epitopes along their amino acid sequences, both lectins share strong identities/homologies with the epitope-containing regions of other legume lectins, especially the two-chain lectins LcA and PsA (see Figure 8 for explanation).

#### 2.1.2. Homodimeric Two-Chain Lectins

**Figure 6:**
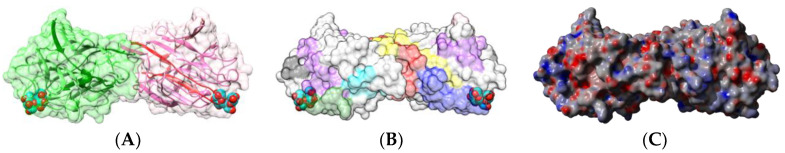
(**A**). Three-dimensional structure of the lentil lectin LcA, showing the dimeric arrangement of the lectin monomers (PDB code 1LES). Secondary structure features of the heavy (pale color) and light (hard color) subunits are represented and the molecular surface is shown in transparency. Monomers I and II are colored green and pink, respectively. Sucrose molecules complexed to the carbohydrate-binding sites in each monomer are colored cyan. (**B**). Localization of the sequential IgE-binding epitopes identified at the molecular surface of the LcA homodimer. Epitopes 1, 2, 5, 6, 7, 8, and 9 are colored red (1), blue (2), green (5), grey (6), purple (7), cyan (8), and yellow (9), respectively. In each monomer, epitopes 1–7 are located on heavy subunits while epitopes 8 and 9 occur in light subunits. (**C**). Electrostatic potentials calculated at the surface of LcA. (**A**,**B**) are not centered.

**Figure 7:**
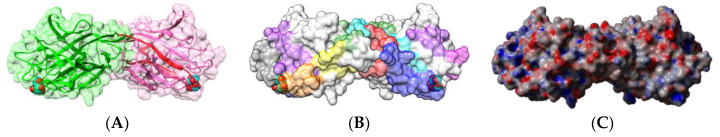
(**A**). Three-dimensional structure of the garden pea lectin PsA, showing the dimeric arrangement of the lectin monomers (PDB code 1BQP). Secondary structure features of the heavy (pale color) and light (hard color) subunits are represented and the molecular surface is shown in transparency. Monomers I and II are colored green and pink, respectively. Mannose molecules complexed to the carbohydrate-binding sites in each monomer are colored cyan. (**B**). Localization of the sequential IgE-binding epitopes identified at the molecular surface of the PsA homodimer. Epitopes 1–10 are colored red (1), blue (2), green 35), magenta (4), grey (5), orange (6), purple (7), brown (8), yellow (9), and cyan (10) respectively. In each monomer, epitopes 1–7 are located on heavy subunits while epitopes 8–10 occur in light subunits. (**C**). Electrostatic potentials calculated at the surface of PsA. (**A**,**B**) are not centered.

Two homodimeric two-chain lectins have been investigated with respect to their IgE-binding activity, namely the lentil (*Lens culinaris*) lectin LcA and the garden pea (*Pisum sativum*) lectin PsA [28,29,62]. Both lectins consist of two protomers with a canonical β-sandwich structure, associated by non-covalent bonds in a homodimer which possesses two CBS located at both ends of the dimer (Figure 6A and Figure 7A). The CBSs readily accommodate mannose, the trimannoside core of *N*-acetyllactosaminic *N*-glycans, and more complex high-mannose glycans.

Nine sequential B-epitopes have been identified at the surface of both the β-subunit (7) and α-subunit (2), in each protomer of lentil lectin LcA (Figure 6B). Similarly, 10 sequential B-epitopes were characterized at the surface of the β-subunit (8) and α-subunit (2) of each garden pea lectin PsA protomer (Figure 7B) [17].

**Figure 8:**
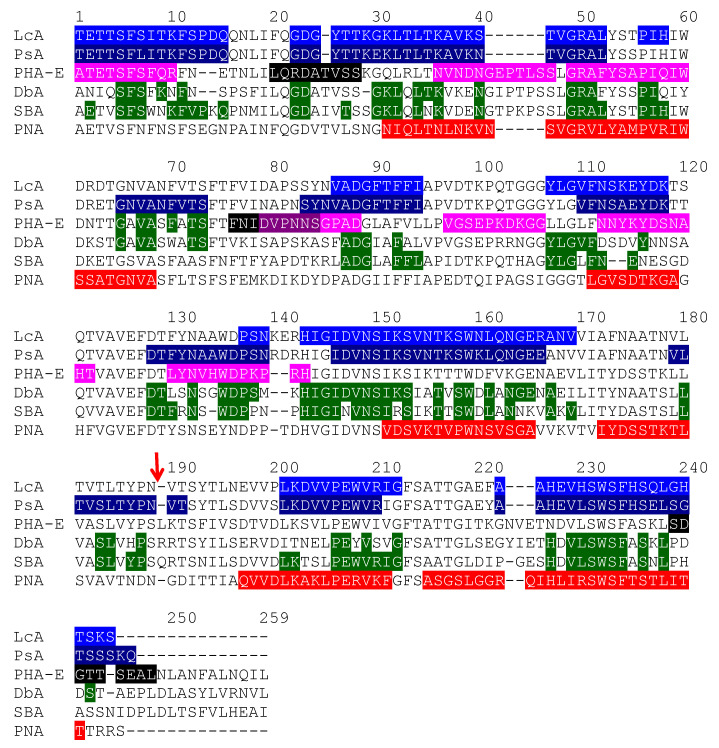
Multiple amino acid sequence alignment of two-chain (LcA, PsA) and single-chain (PHA-E, DbA, SBA, PNA) lectins, showing the localization of B- and T-epitopes along the amino acid sequences. The IgE-binding B-epitopic regions are indicated in white letters highlighted blue (LcA), deep blue (PsA), magenta (PHA-E), and red (PNA), respectively. Regions of DbA and SBA homologous of B-epitopic regions of LcA and PsA are indicated in white letters highlighted green. The three T-epitopes predicted along the amino acid sequence of PHA-E are shown in white letters highlighted black and burgundy color for T-epitope #2 overlapping the B-epitope #4 region.

Due to the high sequential and structural homologies occurring among the closely-related members of the legume lectin family, most of the identified sequential IgE-binding B-epitopes exhibit a very similar positioning along the amino acid sequence and at the surface of the lectin protomers, in both homotetrameric single-chain lectins (PNA, PHA-E, DbA, SBA) and homodimeric two-chain lectins (LcA, PsA), irrespective of their structural architecture and carbohydrate-binding specificities (Figure 8). Most sequences encoding lectins that are abundant in other edible legumes, such as lupine (*Lupinus albus*), faba bean (*Vicia faba*), winged bean (*Psophocarpus teragonolobus*), azuki bean (*Vigna angularis*), and many others, look very similar to those of LcA from lentil, PsA from pea, PNA from peanut, and PHA from kidney bean and, especially, at the location of some of the predicted IgE-binding epitopes. Accordingly, they are suspected to behave as potential allergens even though at present no evidence exists to support their allergenicity.

**Figure 9:**
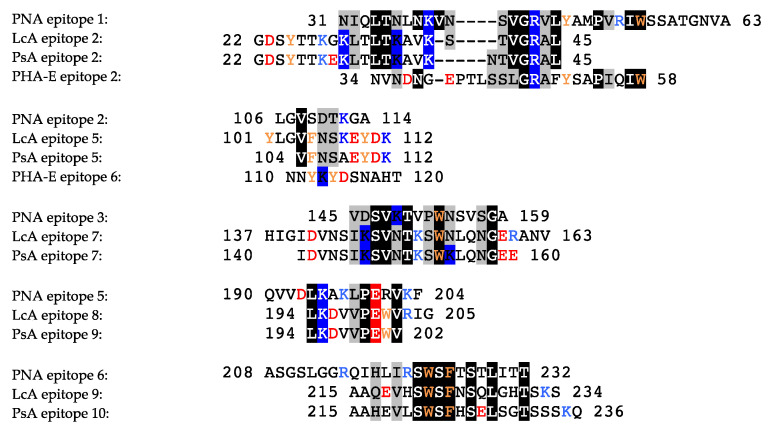
Comparison of some sequential IgE-binding epitopes identified at homologous positions along the amino acid sequence of PNA, LcA, and PsA. Identical residues are in boxed white letters. Structurally homologous residues according to Risler’s matrix [63] are grey boxed. Positively (K,R) and negatively (D,E) charged residues are colored blue and red, respectively. Aromatic residues (F,Y,W) are colored orange.

In addition, most of these predicted IgE-binding epitopic regions contain a high proportion of either electropositively (R,K) or electronegatively (D,E) charged residues (Figure 9), which explains the high electrostatic potentials often calculated for these IgE-binding regions at the molecular surfaces of both single- and two-chain legume lectins (Figure 1C, Figure 2C, Figure 4C, Figure 5C, Figure 6C and Figure 7C). Aromatic residues (F,Y,W) also frequently occur in the IgE-binding epitopic regions common to single- and two-chain legume lectins.

**Figure 10:**
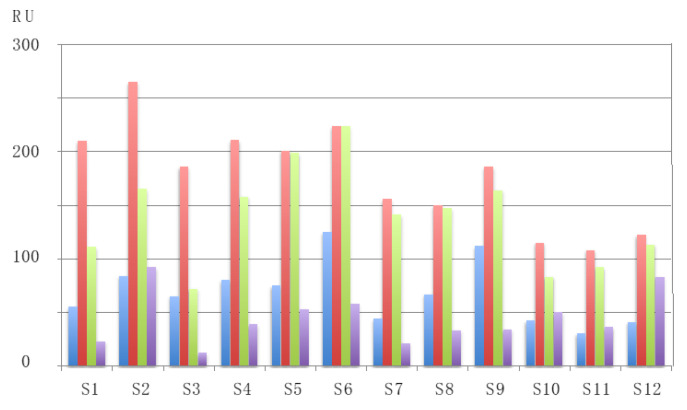
SPR measurement, expressed as resonance units (RU), of the interaction of sera (S1–S12) from peanut-allergic patients with PNA (blue color), LcA (red color), PsA (green color), and PHA-E (violet color). Each value is the mean of three independent measurements [17].

Obviously, these B-epitope similarities account for their IgE-binding cross-reactivity observed among different legume lectins, as previously reported for sera from peanut-allergic patients [17]. As shown from surface plasmon resonance (SPR) measurements, both PNA, PHA-E, LcA, and PsA lectins, readily interacted with all of the probed sera from peanut-allergic patients (Figure 10). However, a stronger IgE-binding cross-reactivity was observed for two-chain legume lectins, LcA and PsA, compared to that observed for single-chain legume lectins, PNA and PHA-E. These discrepancies among the single- and two-chain lectins, would depend on the way the protomers are assembled in both types of lectins, the dimeric association of protomers favoring a better exposure of epitopes, compared to the tetrameric association in which some epitopes become buried at the protomer interfaces.

**Figure 11:**
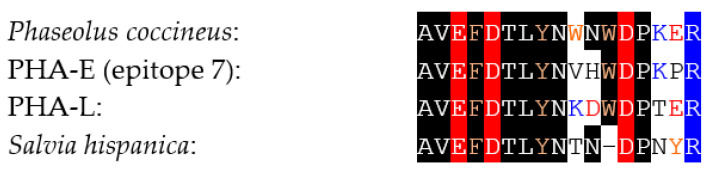
Comparison of the amino acid sequence stretch of Salvia hispanica with the corresponding regions of PHA-E, PHA-L and Phaseolus coccineus lectin.

Another homodimeric two-chain lectin from chia (*Salvia hispanica*) seeds has been identified as a potential IgE-binding allergen [46]. The single amino acid sequence stretch AVEFDTLYNTNDPNYR characterized by mass spectrometry for this IgE-binding allergen exhibits a high percentage of identity with the *Phaseolus coccineus* (81.25%) and the *Phaseolus vulgaris* PHA-E (75.0%) and PHA-L (75.0%) lectins (Figure 11):

Sera from people allergic to peanut-allergic people that were available to perform the IgE-binding cross-reactions with different lectins contained specific IgE that primarily recognize the main allergenic proteins from peanut, the vicillin Ara h 1, the 2S albumins Ara h 2 and Ara h 6, and the legumin Ara h 3. Therefore, PNA (also designated as Ara h agglutinin allergen) and other IgE-binding cross-reacting lectins from other legume seeds (LcA, PsA, PHA, SBA), do not actually correspond to the main components of allergies. In this respect, none of the peanut allergies identified so far can be attributed to the PNA allergen.

### 2.2. Type II Ribosome Inactivating Proteins (RIP-II)

Type II Ribosome-Inactivating Proteins (RIP-II) are chimerolectins built from a toxic A-chain with a catalytic RNA *N*-glycosidase activity, covalently associated through a disulfide bridge to a two-domain B-chain with lectin activity [14,15]. The B-chain often exhibits Gal/GalNAc-binding specificity. After recognition of Gal/GalNAc-containing glycans on the cell surface by the B-chain, RIP-II molecules are engulfed into the cell and are subsequently cleaved to liberate the toxic A-chain. Through its RNA *N*-glycosidase activity, the A-chain inactivates the ribosomal 28S RNA via the depurination of an adenine base, which in turn blocks the protein synthesis and provokes cell death [64].

**Figure 12:**
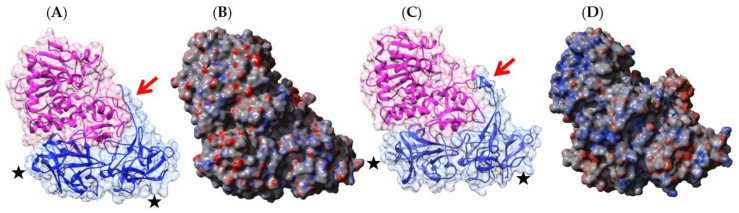
(**A**). Three-dimensional structure of ricin (PDB code 2AAI), showing the arrangement of the catalytic chain A (colored magenta) and the lectin chain B (colored blue), covalently linked by a disulfide bridge (colored red) indicated by a red arrow. Secondary structure features of both chains are represented and their molecular surfaces are shown in transparency. The location of the CBSs in the lectin chain is indicated by a black star (★). (**B**). Electrostatic potentials calculated at the surface of ricin. (**C**). Three-dimensional structure of the modeled SNA-I, showing the arrangement of the catalytic chain A (colored magenta) and the lectin chain B (colored blue), covalently linked by a disulfide bridge (colored red), indicated by a red arrow. Secondary structure features of both chains are represented and their molecular surfaces are shown in transparency. The location of the CBSs in the lectin chain is indicated by a black star (★). (**D**). Electrostatic potentials calculated at the surface of the SNA-I model.

Three RIP-II have been identified as potential food allergens, ricin from castor bean (*Ricinus communis*) seeds, SNA-I from the black elderberry (*Sambucus nigra*) [65,66], and the RIP-II from the dwarf elder (*Sambucus ebulus*) [67]. All these chimerolectins exhibit a similar three-dimensional structure, made of both A- and B-chains covalently linked by a disulfide bridge occurring between two cysteine residues located at the *C*-terminus of the A-chain and at the *N*-terminus of the B-chain, respectively (Figure 12A–D). Toxic lectins from mistletoe (*Viscum album*) also exhibit a similar organization of their A- and B-chains [68,69]. However, since no IgE-binding B-epitope or T-epitope were identified at the surface of these molecules, we do not know exactly what part of these allergens, either the A-chain or the B-chain or both, triggers occupational allergic manifestations in sensitized people.

### 2.3. Wheat Germ Agglutinin (WGA)

**Figure 13:**
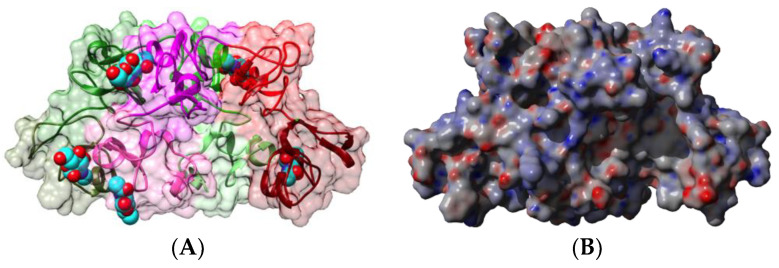
(**A**). Three-dimension al structure of WGA (PDB code 2UVO), showing the arrangement of two hevein tetramers, colored red and green, respectively. Secondary structure features of both hevein units are represented and their molecular surfaces are shown in transparency. N-acetylglucosamine residues (colored cyan) are anchored to the CBSs occurring in some of the hevein units. (**B**). Electrostatic potentials calculated at the surface of WGA. (**A**) is not centered and thus, is truncated.

Wheat germ agglutinin WGA (Tri a 18 allergen), has been identified as a hevein motif-containing lectin because it results from the association of two tetramers, each comprising four tandemly arrayed hevein motifs that specifically bind to GlcNAc via a network of hydrogen bonds and stacking interactions (Figure 13). IgE-binding epitopes of WGA consist of the B-epitopes that have been identified at the surface of the hevein (Hev b 6.02) polypeptide allergen [70,71] (see Section 2.4).

### 2.4. Chitinases Containing a Hevein Domain

**Figure 14:**
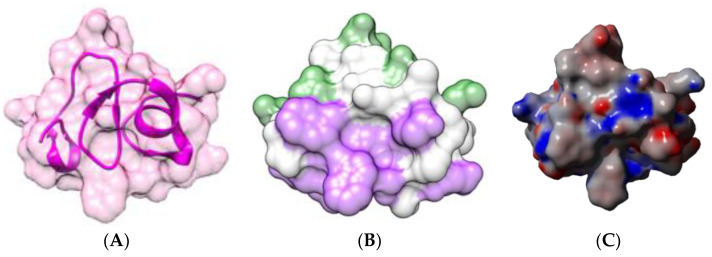
(**A**). Three-dimensional structure of Hev b 6 (PDB code 1WKX), showing the arrangement of the secondary structure features along the short polypeptide chain. The molecular surface is shown in transparency. (**B**). Mapping of discontinuous B-epitopes on the molecular surface of Hev b 6, colored green (epitope 1) and purple (epitope 2), respectively. (**C**). Electrostatic potentials calculated at the surface of hevein. (**A**,**B**) are not centered (and too large) and thus, are truncated.

Hevein occurs as a free polypeptide allergen Hev b 6.02, which has been identified as a major allergen in the latex from the rubber tree (*Ricinus communis*) [35,72,73]. This is a small contact allergen of only 43 amino acids, organized in a central β-hairpin sandwiched between two short *N*- and *C*-terminal α-helices (Figure 14A,C), tightly structured by four disulfide bridges. Two discontinuous B-epitopes have been identified at the molecular surface of Hev b 6.02, corresponding to amino acid residues 2, 10–12, 14–15, 26, 40, 42 (epitope 1) and 5–6, 19–21, 23, 29, 33–34 (epitope 2), that specifically interact with two moAbs scFv G7 and scFv H7, respectively (Figure 14B) [71]. T-epitopes corresponding to HLA-DR4, have been identified on prohevein Hev b 6.01, the precursor of hevein which is further post-transcriptionally cleaved in two Hev b 6.02 (hevein) and Hev b 6.03 proteins, but none of them occurs along the Hev b 6.02 amino acid sequence [74].

**Figure 15:**
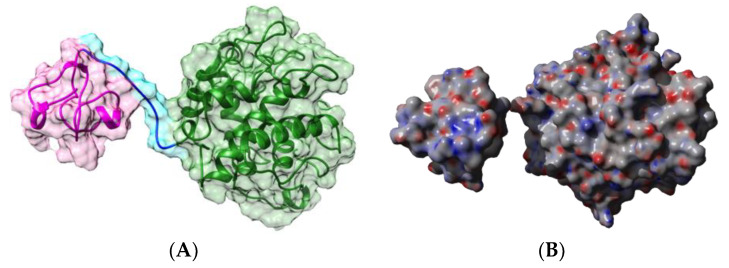
(**A**). Three-dimensional model built for chitinase I allergen Mus a 2 from banana, made of an N-terminal hevein domain (colored magenta) linked by an extended hinge (colored cyan) to the C-terminal chitin-binding glycosyl hydrolase 19 domain (colored green). (**B**). Electrostatic potentials calculated at the surface of the Mus a 2 model.

Hevein occurs as a *N*-terminal domain in various plant endochitinases belonging essentially to classes I and class IV chitinases. Class I chitinase allergens have been identified in banana (Mus a 2) [31,75], chestnut (*Castanea sativa*) (Cas s 5) [76,77], avocado (*Persea americana*) (Per a 1) [36,76,78,79], passion fruit (*Passiflora edulis*) (Pas ed chitinase) [39], kiwi (*Actinidia chinensis*) (Act c chitinase) [37], papaya (*Carica papaya*) (Car p chitinase) [38], custard apple (*Annona cherimola*) [39,80], tomato (*Lycopersicum esculentum*) (Lyc es chitinase) [39,81], and in the rubber tree latex as a contact allergen Hev b 11 [82]. Similar to Mus a 2 from banana, class I chitinases comprise an *N*-terminal hevein domain, linked by an extended hinge region to a *C*-terminal chitin-binding catalytic domain (Figure 15).

Class IV chitinases, which also contain a shortened chitin-binding module, have been identified in corn (*Zea mays*) (Zea m 8) [33] and in grape (*Vitis vinifera*) (Vit v chitinase) [40]. However, the allergenicity of Zea m 8 is not restricted to the chitin-binding hevein domain since the C-terminal catalytic domain expressed as a recombinant molecule in *E. coli* still interacted with sera from patients allergic to maize [33].

Due to their widespread distribution in plants and plant products, chitinases and especially class I chitinases, account for IgE-binding cross-reactivity between various fruits and vegetables [83,84,85].

### 2.5. Jacalin-Related Lectins

**Figure 16:**
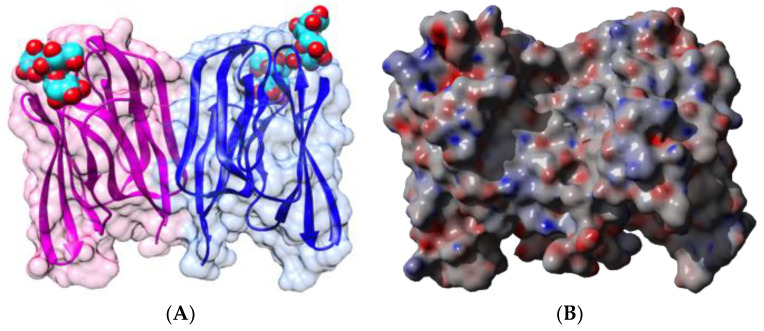
(**A**). Three-dimensional structure of the BanLec dimer (PDB code 4PIT), showing the β-prism II arrangement of the β-sheets in each monomer. Molecular surfaces of both monomers, colored magenta and blue, respectively, are shown in transparency. Dimannoside ligands (colored cyan) anchor to the CBSs located at the top of both monomers. (**B**). Electrostatic potentials calculated at the surface of BanLec. (**A**) is not centered and thus, is truncated.

Jacalin-related lectins consist of a very homogeneous group of plant lectins, exhibiting a β-prism II structure for their constituent protomers, usually organized in either homodimeric or homotetrameric structures displaying two or four identical CBSs, respectively. In this respect, BanLec, the jacalin-related lectin from banana results from the non-covalent association of two protomers with the β-prism II structure. The β-prism II scaffold consists of three bundles of four antiparallel β-strands arranged into a β-prism structure along a longitudinal axis. Each protomer possesses a CBSs located at the top of the β-prism structure, which specifically recognizes *N*-glycans of the high-mannose type (Figure 16) [86].

Banana consumption was reported to elicit IgG4 antibody production [47], and BanLec was also reported to induce a non-specific activation of basophils and mast cells in atopic subjects [87]. In vitro treatment of Balb/c and C57 BL/6 splenocytes with the recombinant BanLec resulted in a proliferation of T cells accompanied by an increased secretion of INFγ [88]. Similarly, the man-specific lectin ArtinM lectin from *Artocarpus heterophyllus*, activated mast cells and induced the release of newly synthesized IL-4 without, however, any co-stimulatory effect on the FcεRI degranulation [89]. Conversely, the Gal/GalNAc-specific jacalin from jackfruit (*Artocarpus integrifolia*), did not induce any release of IL-4, IL-13, and histamine from activated basophils [29]. At present, no data are available on the identification and localization of B- and T-epitopes at the molecular surface of jacalin-related lectin allergens.

### 2.6. GNA-Like Lectins

**Figure 17:**
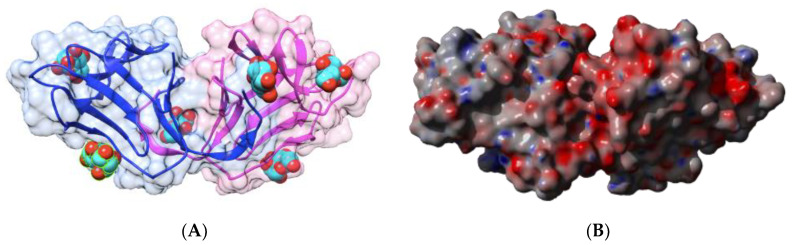
(**A**). Three-dimensional structure of the homodimeric garlic lectin ASA (PDB code 1KJ1), showing the arrangement of both monomers, colored magenta and blue, respectively, through a reciprocal swapping of their C-terminal ends. Secondary structure features of both monomers are represented and their molecular surfaces are shown in transparency. Mannose residues (colored cyan) are anchored to the three CBSs occurring in each monomer. (**B**). Electrostatic potentials calculated at the surface of ASA.

Food allergic manifestations associated with the consumption of spices like garlic (*Allium sativum*) and onion (*Allium cepa*) have been attributed to the occurrence of lectins belonging to the vast group of GNA-like lectins in these edible bulbs [41,42,90,91,92]. Being members of the GNA-related lectin group, both the garlic lectin ASA and the onion ACA lectin possess protomers exhibiting the β-prism I fold, which consists of three bundles of four antiparallel β-strands arranged into a β-prism structure perpendicular to the axis. Each protomer contains three CBSs which specifically recognize mannose residues. Two non-covalently linked protomers build the hexavalent homodimeric structure found in the garlic lectin ASA (Figure 17).

In spite of their allergenic properties, no B- and T-epitope of garlic and onion GNA-related lectins have been characterized. In contrast to the garlic and onion lectins, the closely-related taro (*Colocasia esculenta*) lectin was not identified as a food allergen [93].

### 2.7. Nictaba-Related Lectins

**Figure 18:**
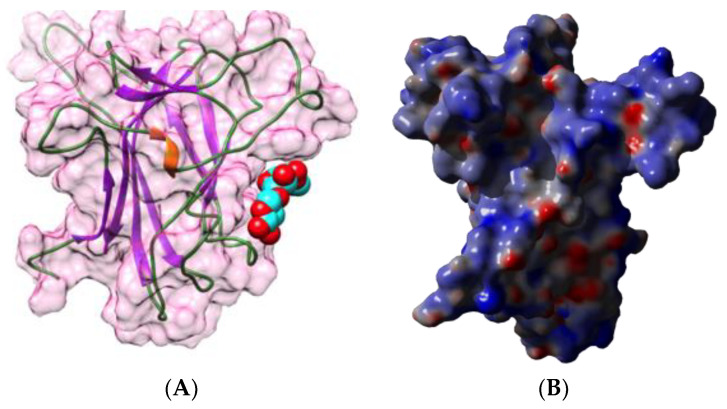
(**A**). Three-dimensional model built for the 17 kDa Nictaba-related lectin from *Cucumis melo*, showing the β-sheets arranged in a β-sandwich structure. Secondary structure features are represented and the molecular surface is shown in transparency. GlcNAc residues anchored to the possible CBS are colored cyan. (**B**). Electrostatic potentials calculated at the surface of the model built for the Nictaba-related lectin from *Cucumis melo*. (**A**) is too large and not centered, thus it is truncated.

The 17kDa protein in the phloem sap of watermelon (*Cucumis melo*) belongs to the Nictaba-related lectins and has been identified as an allergen involved in systemic reactions to melon and watermelon consumption [48]. Similar to the tobacco (*Nicotiana tabacum*) lectin Nictaba, the protein is exclusively composed of β-stands arranged in a β-sandwich structure, containing a single CBS with a GlcNAc-specificity (Figure 18) [94]. Currently, there are no investigations on the occurrence of B- and T-epitopes in these molecules.

## 3. Discussion

The group of plant lectins comprise a huge number of proteins which share the common property to specifically recognize carbohydrate structures, in spite of very different amino acid sequences and structural scaffolds. According to their carbohydrate-binding domain, plant lectins have been divided into twelve distinct families (Table 3), which display similar or different carbohydrate-binding specificities towards simple and complex sugars.

Lectins with allergenic properties have been identified in 6 out of 12 plant lectin families, including the GNA family, the hevein family, the jacalin-related lectin (JRL) family, the legume lectin family, the Nictaba family, and the ricin B family. However, only a restricted number of 8 lectins comprising Mus a 2 from banana, Tri a 18 from wheat, Zea m 8 from corn, Bra r 2 from turnip, Cas s 5 from chestnut, the contact allergens Hev b 6 and Hev b 11 from the rubber tree, and Pers a 1 from avocado, categorize as WHO-IUIS-approved food allergens [27]. Other identified lectin allergens reported in Table 1, have not yet been officially assigned as food allergens in spite of their IgE-binding ability and capacity to trigger some Th2 cytokin/interleukin response in atopic or sensitized people. In addition, other lectins from non-edible plants, such as Con A from jackbean (*Canavalia ensiformis*), DsA from jimson weed (*Datura stramonium*), and ricin from the castor bean (*Ricinus communis*), have also been recognized as potential inducers of interleukin responses [29].

Most of the plant lectins identified as potential food allergens (19 over 26) belong to two families, in particular the family of legume lectins (7 out of 26 proteins) and the group of chitinases with a hevein domain (12 out of 26 proteins) (Table 2). Legume lectin allergens like the lectins from peanut (Ara h agglutinin/PNA), kidney bean (PHA-E, PHA-L), soybean (SBA), horse gram (DbA), lentil (LcA), and pea (PsA), occur as seed storage proteins sequestered in protein bodies, together with the major cupin allergens, 7S globulins/vicilins and 11S globulins/legumins, and 2S albumin allergens [96]. However, similar to Ara h agglutinin/PNA of peanut and SBA of soybean, they most probably consist of minor allergens [43]. In contrast, class I chitinases are important allergens in many fruits, responsible for oral syndromes often associated with IgE-binding cross-reactivity between different fruits, e.g., between avocado, banana, kiwi, and chestnut [67,68,69]. In addition to allergenic chitinases of types I and IV, which both possess a hevein domain, other allergenic chitinases devoid of hevein domain have been identified in various plant species including Cof a 1 from coffee (*Coffea arabica*), Hev b 11 and hevamine from the rubber tree *Hevea brasiliensis*, Pun g 14 from pomegranate (*Punica granatum*), Trip s 1 from *Triplochiton scleroxylon*, Zea m 8 from maize (*Zea mays)*, and Ziz m 1 from *Ziziphus mauritiana* (http://allergome.org). Obviously, their allergenicity is not attributed to the hevein domain, which reinforces the idea that the allergenicity displayed by chitinases containing a hevein domain could also result from the enzyme part of these complex proteins. Similarly, it is predictable that some allergenicity displayed by type II RIPs such as SNA-I also depends on the A domain responsible for the *N*-glycosidase activity of these complex proteins.

To date, however, the immunogenicity of lectins as allergenic proteins remains poorly known and difficult to foresee, because of the lack of information on the T-cell epitopes in these proteins. Except for PHA-E from black turtle bean [59], no information on the number and distribution of T epitopes is available for other potentially allergenic lectins. However, some information has been collected on the location and distribution of IgE-binding B-cell epitopic regions on the surface of a few lectins, especially legume lectins. In this respect, legume lectins exhibit a B-cell epitope density and diversity comparable to those of other plant allergenic proteins.

Even though plant lectins have been identified as relevant allergens, it is currently not possible to investigate lectins of plant origin as potential food allergens, because except for rHev b 6.02 (recombinant hevein), none of these proteins, e.g., PNA for peanut allergy diagnosis, are available in natural or recombinant form in the allergen microarrays commercially available for the allergen component-resolved diagnosis of food allergy. In addition, the ability of many plant lectins to specifically recognize the carbohydrate moiety of IgE antibodies could generate some unexpected false-positive results, irrespective of the allergic status of the patients [97,98]. Similarly, many plant lectins, e.g., PHA from kidney bean [28] and ArtinM from jackfruit (*Artocarpus heterophyllus*) [89], can non-specifically degranulate both T lymphocytes and mast cells using an IgE-independent mechanism, and thus interfere with the specific degranulation of IgE-activated cells. To overcome these possible sources of bias, it is absolutely necessary to perform the analyses in the presence of a cocktail of inhibitory sugars, e.g., Man + Gal + Fuc, capable to prevent the non-specific binding of lectins to glycan structures associated with IgE and the cell surface of T-lymphocytes or mast cells.

Due to their widespread distribution in edible seeds and fruits, legume lectins and class I chitinases should be investigated in legume seed allergies (peanut, kidney bean, soybean, lentil, pea) and fruit allergies (banana, avocado, mango, kiwi, chestnut), using natural or recombinant lectins as molecular probes, in addition to rHev b 6.02, which is currently available for the IgE-mediated allergy diagnosis.

## 4. Bioinformatics

Multiple amino acid sequence comparisons were performed with the CLUSTAL-X program [99].

The atomic coordinates of lectins including PNA from peanut *Arachis hypogaea* (PDB code 2PEL) [100], DbA from horse gram *Dolichos biflorus* (PDB code 1LU1) [101], SBA from soybean *Glycine max* (PDB code 2SBA) [102], LcA from lentil *Lens culinaris* (PDB code 1LES) [103], ricin from castor bean *Ricinus communis* (PDB code 2AAI) [104], WGA from wheat *Triticum aestivum* (PDB code 2UVO) [105], Hev b 6 from the rubber tree *Hevea brasiliensis* (PDB code 1WKX), BanLec from banana Musa *acuminata* (PDB code 4PIT) [86], and ASA from garlic *Allium sativum* (PDB code 1KJ1) [106], were taken from the Protein Data Bank (http://www.rcsb.org/pdb/) [107].

Homology modeling of other lectins including PHA-E from the black turtle bean *Phaseolus vulgaris* [Acces. AHB17899.1], type II RIP SNA-I from the black elderberry *Sambucus nigra* [Acces. Q41358.1], the chitinase I Mus a 2 from banana *Musa acuminata* [Acces. XM_009394597.2], and the Nictaba-related lectin from watermelon *Cucumis melo* [Acces. NM_001305649.1], was performed with the YASARA Structure program [108] using various protein templates from the PDB, depending on the overall structural scaffold to which they belong (e.g., 1G8W from *Phaseolus vulgaris* [109], 1LU1 from *Dolichos biflorus* [101], 3UL2 from *Dolichos lablab* [110], 3WCS [111] and 5AVA [112] from *Phaseolus vulgaris* (PHA-E) for modeling the black turtle bean from *Phaseolus vulgaris* [59], shown in Figure 2 and Figure 3). PROCHECK [113], ANOLEA [114], and the calculated QMEAN scores [115,116] were used to assess the geometric and thermodynamic qualities of the three-dimensional models.

The surface-exposed electrostatic potentials of legume lectins were calculated and displayed (negatively and positively charged regions colored red and blue, respectively, and neutral regions colored grey) at the molecular surface with YASARA, using the classic values for the inner and outer dielectric constants applied to the proteins and the solvent, fixed at 4.0 and 80.0, respectively.

Molecular cartoons were drawn with Chimera [117] and YASARA.

## Figures and Tables

**Table 1:** Lectin content of some edible plants, compared to their protein content.

Edible Plant	Lectin	Lectin Content (% of Total Protein)	Ref.
Lentil (*Lens culinaris*)	LcA	2.5%	[18]
Pea (*Pisum sativum*)	PsA	2.5%	[19]
Soybean (*Glycine max*)	SBA	2%	[20]
Peanut (*Arachis hypogaea*)	PNA	1.5%	[21]
Horse gram (*Dolichos biflorus*)	DbA	10%	[22]
Kidney bean (*Phaseolus vulgaris*)	PHA	1%	[23]
Wheat (*Triticum aestivum*)	WGA	3%	[24]
Garlic (*Allium sativum*)	ASA	10%	[25]
Black elderberry (*Sambucus nigra*)	SNA-I	3%	[25]
Banana (*Musa acuminata*)	Mus a 2	2.3%	[26]

**Table 2:** List of lectins identified as potential food allergens. Lectin allergens approved by the International Union of Immunological Societies (WHO/IUIS Allergen Nomenclature Sub-Committee) [27], are indicated. Lectin allergens Hev b 6 and Hev b 11 from Castor bean (*Ricinus communis*) latex, are responsible for contact allergies but also for IgE-binding cross-reactivity with hevein-containing food chitinase proteins.

Allergen	Protein	Plant Species	IUIS Approved	Food Allergen	Ref.
Mus a 2	Class I chitinase	*Musa acuminata*	+	+	[31]
Tri a 18	Wheat germ agglutinin	*Triticum aestivum*	+	+	[32]
Zea m 8	Class IV chitinase	*Zea mays*	+	+	[33]
Bra r 2	Chitin-binding protein	*Brassica rapa*	+	+	[34]
Cas s 5	Chitinase	*Castanea sativa*	+	+	*
Hev b 6	Hevein precursor	*Hevea brasiliensis*	+	Contact	[35]
Hev b 11	Class I chitinase	*Hevea brasiliensis*	+	Contact	*
Pers a 1	Class I chitinase	*Persea americana*	+	+	[36]
Act c chitinase	Class I chitinase	*Actinidia chinensis*		+	[37]
Car p chitinase	Class I chitinase	*Carica papaya*		+	[38]
Lyc es chitinase	Class I chitinase	*Lycopersicum esculentum*		+	[39]
Ann ch chitinase	Class I chitinase	*Annona cherimola*		+	[39]
Pas ed chitinase	Class I chitinase	*Passiflora edulis*		+	[39]
Vit v chitinase	Class IV chitinase	*Vitis vinifera*		+	[40]
AcA	GNA-like Lectin	*Allium cepa*		+	[41]
AsA	GNA-like lectin	*Allium sativum*		+	[42]
Ara h agglutinin	Legume lectin	*Arachis hypogaea*		+	[43]
DbA	Legume lectin	*Dolichos biflorus*		+	[44]
SBA	Legume lectin	*Glycine max*		+	[45]
LcA	Legume lectin	*Lens culinaris*		+	[29]
PHA-E	Legume lectin	*Phaseolus vulgaris*		+	[29]
PHA-L	Legume lectin	*Phaseolus vulgaris*		+	[29]
PsA	Legume lectin	*Pisum sativum*		+	[29]
Chia lectin	Legume lectin	*Salvia hispanica*			[29]
Ricin	RIP-II	*Ricinus communis*		+	[46]
SNA-I	RIP-II	*Sambucus nigra*		+	[29]
BanLec	Jacalin-related lectin	*Musa acuminata*		+	[47]
Cuc m 17 kDa	Nictaba-related lectin	*Cucumis melo*		+	[48]

* No references available at the WHO/IUIS Allergen Nomenclature Sub-Committee. + means yes.

**Table 3:** Classification of plant lectins in twelve families. Families containing allergens are in bold.

Lectin Family	Structural Scaffold	Simple Sugar Specificity	Complex Sugar Specificity
*Agaricus bisporus* lectin family	β-sandwich	Gal/GalNAc	T-antigen
Amaranthin family	β-trefoil	Gal/GalNAc	T-antigen
Class V chitinase homolog family	TIM-barrel (α_8_β_8_)	Man	High-mannose glycans
Cyanivirin family	β-barrel	Man	High-mannose glycans
*Euonymus europaeus* lectin family	β-trefoil	Gal, Man	B + H group + high-mannose glycans
GNA-like family	β-trefoil	Man	High-mannose glycans
Hevein family	hevein domain	GlcNAc	chitin
Jacalin-related lectin family	β-barrel	Gal, GalNAc, Man	T-antigen, High-mannose glycans
Legume lectin family	β-sandwich	Gal, GalNAc, Man	Complex glycans, High-mannose glycans
Lysin motif domain family	αβ	GlcNAc	Chitin, lipochito- oligosaccharides,
Nictaba family	β-sandwich	GlcNAc, Man	Chitooligosaccharides, high-mannose glycans, complex glycans
Ricin B family *	β-trefoil	Gal	Lactose

* SNA-I, the RIP-II isolated from *Sambucus nigra* bark, exhibits an unusual Neu5Acα2,6-Gal/GalNAc- binding specificity [95].

## Abbreviations

ACA	*Allium cepa* (onion) agglutinin/lectin
ASA	*Allium sativum* (garlic) agglutinin/lectin
BanLec	Banana (*Musa acuminata*) lectin
CBS	Carbohydrate-binding site
DbA	*Dolichos biflorus* (horse gram) agglutinin/lectin
GNA	*Galanthus nivalis* (snowdrop) agglutinin/lectin
IgE	Immunoglobulin E
INFγ	Interferon-gamma
LcA	*Lens culinaris* (lentil) agglutinin/lectin
moAb	Monoclonal antibody
Nictaba	*Nicotiana tabacum* (tobacco) agglutinin/lectin
PHA-E	Erythroagglutinin of *Phaseolus* (kidney bean) agglutinin/lectin
PHA-L	Leucoagglutinin of *Phaseolus* agglutinin/lectin
PNA	Peanut (*Arachis hypogaea*) agglutinin/lectin (also known as Ara h agglutinin)
PsA	*Pisum sativum* (garden pea) agglutinin/lectin
RU	Resonance unit
SBA	Soybean (*Glycine max*) agglutinin/lectin
SNA	*Sambucus nigra* (black elderberry) agglutinin/lectin
SPR	Surface plasmon resonance
WGA	Wheat (*Triticum aestivum*) germ agglutinin/lectin (also known as Tri a 18)

## References

[1] Sicherer S.H., Sampson H.A. (2018). Food allergy: A review and update on epidemiology, pathogenesis, diagnosis, prevention, and management. J. Allergy Clin. Immunol..

[2] Iweala O.I., Choudhary S.K., Commins S.P. (2018). Food allergy. Curr. Gastroenterol. Rep..

[3] Sathe S.K., Sharma G.M. (2009). Effects of food processing on food allergens. Mol. Nutr. Food Res..

[4] Breiteneder H., Radauer C. (2004). A classification of plant food allergens. J. Allergy Clin. Immunol..

[5] Breiteneder H., Mills E.N. (2005). Molecular properties of food allergens. J. Allergy Clin. Immunol..

[6] Lorenz A.R., Scheurer S., Vieths S. (2015). Food allergens: Molecular and immunological aspects, allergen databases and cross-reactivity. Chem. Immunol. Allergy.

[7] Barre A., Sordet C., Culerrier R., Rancé F., Didier A., Rougé P. (2008). Vicilin allergens of peanut and tree nuts (walnut, hazelnut and cashew nut) share structurally related IgE-binding epitopes. Mol. Immunol..

[8] Leoni C., Volpicella M., Dileo M.C.G., Gattulli B.A.R. (2019). Chitinases as food allergens. Molecules.

[9] Azofra García J., Cuesta-Herranz J., Perea Lam N., Díaz-Perales A. (2014). Anaphylaxis mediated by thaumatin-like proteins. J. Investig. Allergol. Clin. Immunol..

[10] Radauer C., Lackner P., Breiteneder H. (2008). The Bet v 1 fold: An ancient, versatile scaffold for binding of large, hydrophobic ligands. BMC Evol. Biol..

[11] Bienvenu F., Barre A., Viel S., Garnier L., Guyon C., Favre-Metz C., Pauli G., Bienvenu J., Rougé P. (2013). Are defensins important plant allergens?. Rev. Fr. Allergol..

[12] Pascal M., Muñoz-Cano R., Reina Z., Palacín A., Vilella R., Picado C., Juan M., Sánchez-López J., Rueda M., Salcedo G. (2012). Lipid transfer protein syndrome: Clinical pattern, cofactor effect and profile of molecular sensitization to plant-foods and pollens. Clin. Exp. Allergy.

[13] Ladics G.S., Budziszewski G.J., Herman R.A., Herouet-Guicheney C., Joshi S., Lipscomb E.A., McClain S., Ward J.M. (2014). Measurement of endogenous allergens in genetically modified soybean-short communication. Regul. Toxicol. Pharmacol..

[14] Van Damme E.J.M., Peumans W.J., Barre A., Rougé P. (1998). Plant lectins: A composite of several distinct families of structurally and evolutionary related proteins with diverse biological roles. Crit. Rev. Plant Sci..

[15] Peumans W.J., Van Damme E.J.M., Barre A., Rougé P. (2001). Classification of plant lectins in families of structurally and evolutionary related proteins. Adv. Exp. Med. Biol..

[16] Barre A., Bourne Y., Van Damme E.J.M., Rougé P. (2019). Overview of the structure-function relationships of mannose-specific lectins from plants, algae and fungi. Int. J. Mol. Sci..

[17] Rougé P., Culerrier R., Granier C., Rancé F., Barre A. (2010). Characterization of IgE-binding epitopes of peanut (*Arachis hypogaea*) PNA lectin allergen cross-reacting with other structurally related legume lectins. Mol. Immunol..

[18] Fialová D., Tichá M., Kocourek J. (1975). Studies on phytohemagglutinins XXVI. A new type of a lentil hemagglutinin isolated from *Lens esculenta* Moench., subsp. *Microsperma* (Baumg.) barulina. Biochim. Biophys. Acta.

[19] Rougé P. (1977). Biologie des Hémagglutinines chez le Pois (*Pisum sativum* L.). Ph.D. Thesis.

[20] Trembley R., Feng M., Menassa R., Huner N.P.A., Jevnikar A.M., Ma S. (2011). High-yield expression of recombinant soybean agglutinin in plants using transient and stable systems. Transgenic Res..

[21] Lotan R., Skutelsky E., Danon D., Sharon N. (1975). The purification, composition, and specificity of the anti-T lectin from peanut (*Arachis hypogaea*). J. Biol. Chem..

[22] Etzler M.E., Van Driessche E., Rougé P., Beeckmans S., Bøg-Hansen T.C. (1996). The *Dolichos biflorus* lectin family: A model system for studying legume lectin structure and function. Lectins, Biology-Biochemistry, Clinical Biochemistry.

[23] Rigas D.A., Osgood E.E. (1955). Purification and properties of the phytohemagglutinin of *Phaseolus vulgaris*. J. Biol. Chem..

[24] LeVine D., Kaplan M.J., Greenaway P.J. (1972). The purification and characterization of wheat germ agglutinin. Biochem. J..

[25] Peumans W.J., Van Damme E.J.M., Van Driessche E., Franz H., Beeckmans S., Pfüller U., Kallikorm A., Bøg-Hansen T.C. (1993). The role of lectins in the defense of plants. Lectins, Biology-Biochemistry, Clinical Biochemistry.

[26] Nikolic J., Mrkic I., Grozdanovic M., Popovic M., Petersen A., Jappe U., Gavrovic-Jankulovic M. (2014). Protocol for simultaneous isolation of three important banana allergens. J. Chromatogr. B.

[27] Pomés A., Davies J.M., Gadermaier G., Hilger C., Holzhauser T., Lidholm J., Lopata A.L., Mueller G.A., Nandy A., Radauer C. (2018). WHO/IUIS allergen nomenclature: Providing a common language. Mol. Immunol..

[28] Shibasaki M., Sumazaki R., Isovama S., Takita H. (1992). Interaction of lectins with human IgE: IgE-binding property and histamine-releasing activity of twelve plant lectins. Int. Arch. Allergy Immunol..

[29] Haas H., Falcone F.H., Schramm G., Haisch K., Gibbs B.F., Klaucke J., Pöppelmann M., Becker W.-M., Gabius H.-J., Schlaak M. (1999). Dietary lectins can induce in vitro release of IL-4 and IL-13 from human basophils. Eur. J. Immunol..

[30] Moreno A.N., Jamur M.C., Oliver C., Roque-Barreira M.C. (2003). Mast cell degranulation induced by lectins: Effect on neutrophil recruitment. Int. Arch. Allergy Immunol..

[31] Sànchez-Monge R., Blanco C., Díaz-Perales A., Collada C., Carrillo T., Aragoncillo C., Salcedo G. (1999). Isolation and characterization of major banana allergens: Identification as fruit class I chitinases. Clin. Exp. Allergy.

[32] Sutton R., Skerritt J.H., Baldo B.A., Wrigley C.W. (1984). The diversity of allergens involved in baker’s asthma. Clin. Allergy.

[33] Volpicella M., Leoni C., Fanizza I., Distaso M., Leoni G., Farioli L., Naumann T., Pastorello E., Ceci L.R. (2017). Characterization of maize chitinase-A, a tough allergenic molecule. Allergy.

[34] Hänninen A.R., Mikkola J.H., Kalkkinen N., Turjanmaa K., Ylitalo L., Reunala T., Palosuo T. (1999). Increased allergen production in turnip (*Brassica rapa*) by treatments activating defense mechanisms. J. Allergy Clin. Immunol..

[35] Bernstein D.I., Biagini R.E., Karnani R., Hamilton R., Murphy K., Bernstein C., Arif S.A., Berendts B., Yeang H.Y. (2003). In vivo sensitization to purified *Hevea brasiliensis* proteins in health care workers sensitized to natural rubber latex. J. Allergy Clin. Immunol..

[36] Sowka S., Hsieh L.S., Krebitz M., Akasawa A., Martin B.M., Starrett D., Peterbauer C.K., Scheiner O., Breiteneder H. (1998). Identification and cloning of Pers a 1, a 32-kDa endochitinase and major allergen of avocado, and its expression in yeast *Pichia pastoris*. J. Biol. Chem..

[37] Bublin M., Mari A., Ebner C., Knulst A., Scheiner O., Hoffmann-Sommergruber K., Breiteneder H., Radauer C. (2004). IgE sensitization profiles toward green and gold kiwifruits differ among patients allergic to kiwifruit from 3 European countries. J. Allergy Clin. Immunol..

[38] Chen Y.T., Hsu L.H., Huang I.P., Tsai T.C., Lee G.C., Shaw J.F. (2007). Gene cloning and characterization of a novel recombinant antifungal chitinase from papaya (*Carica papaya*). J. Agric. Food Chem..

[39] Díaz-Perales A., Collada C., Blanco C., Sanchez-Monge R., Carrillo T., Aragoncilllo C., Salcedo G. (1999). Cross-reactions in the latex-fruit syndrome: A relevant role of chitinase but not of complex asparagine-linked glycans. J. Allergy Clin. Immunol..

[40] Pastorello E.A., Farioli L., Pravettoni V., Ortolani C., Fortunato D., Giuffrida M.G., Perono Garoffo L., Calamari A.M., Brenna O., Conti A. (2003). Identification of grape and wine allergens as an endochitinase 4, a lipid-transfer protein, and a thaumatin. J. Allergy Allergy Clin. Immunol..

[41] Albanesi M., Pasculli C., Giliberti L., Rossi M.P., Di Bona D., Caiaffa M.F., Macchia L. (2019). Immunological characterization of onion (*Allium cepa*) allergy. Adv. Dermatol. Allergol..

[42] Clement F., Pramod S.N., Venkatesh Y.P. (2010). Identity of the immunomodulatory proteins from garlic (*Allium sativum*) with the major garlic lectins or agglutinins. Int. Immunopharmacol..

[43] Burks A.W., Cockrell G., Connaughton C., Guin J., Allen W., Helm R.M. (1994). Identification of peanut agglutinin and soybean trypsin inhibitor as minor legume allergens. Int. Arch. Allergy Immunol..

[44] Pramod S.N., Krishnakantha T.P., Venkatesh Y.P. (2006). Effect of horse gram lectin (*Dolichos biflorus* agglutinin) on degranulation of mast cells and basophils of atopic subjects: Identification as an allergen. Int. Immunopharmacol..

[45] Natarajan S., Xu C., Bae H., Caperna T.J., Garrett W.M. (2006). Proteomic analysis of allergen and antinitritional proteins in wild and cultivated soybean seeds. J. Plant Biochem. Biotechnol..

[46] García Jiménez S., Pastor Vargas C., de la Heras M., Sanz Matoto A., Vivanco F., Sastre J. (2015). Allergen characterization of chia seeds (*Salvia hispanica*), a new allergenic food. J. Investig. Allergol. Clin. Immunol..

[47] Koshte V.L., Aalbers M., Calkhoven P.G., Aalberse R.C. (1992). The potent IgG4-inducing antigen in banana is a mannose-binding lectin, BanLec-I. Int. Arch. Allergy Immunol..

[48] González-Mancebo E., López-Torrejón G., González de Olano D., Santos S., Gandolfo-Cano M., Meléndez A., Salcedo G., Cuesta-Herranz J., Vivanco F., Pastor-Vargas C. (2010). Identification of potential allergens involved in systemic reactions to melon and watermelon. Ann. Allergy Asthma Immunol..

[49] Van Damme E.J.M., Rougé P., Peumans W.J., Kamerling J.P., Boons G.J., Lee Y.C., Suzuki A., Taniguchi N., Voragen A.G.I. (2007). Plant lectins. Carbohydrate-protein interactions: Plant Lectins.

[50] Vojdani A. (2015). Immune reactivities to peanut proteins, agglutinins, and oleosins. Antern. Ther. Health Med..

[51] White B.L., Gökce E., Nepomuceno A.I., Muddiman D.C., Sanders T.H., Davis J.P. (2013). Comparative proteomic analysis and IgE binding properties of peanut seed and testa (skin). J. Agric. Food Chem..

[52] Zabel P.L., Noujaim A.A., Shysh A., Bray J. (1983). Radioiodinated peanut lectin: A potential radiopharmaceutical for immunodetection of carcinoma expressing the T antigen. Eur. J. Nucl. Med..

[53] Goldstein I.J., Poretz R.D., Liener I.E., Sharon N., Goldstein I.J. (1986). Isolation, physicochemical characterization, and carbohydrate-binding specificity of lectins. The Lectins, Properties, Functions, and Applications in Biology and Medicine.

[54] Haas H., Herzig K.H., André S., Galle J., Gronow A., Gabius H.-J. (2001). Low-dose intragastric administration of *Phaseolus vulgaris* agglutinin (PHA) does not induce immunoglobulin E (IgE) production in Sprague-Dawley rats. Glycoconj. J..

[55] Rougé P., Culerrier R., Thibeau F., Didier A., Barre A. (2011). A case of severe anaphylaxis to kidney bean: Phaseolin (vicilin) and PHA (lectin) identified as putative allergens. Allergy.

[56] Kasera R., Singh B.P., Lavasa S., Prasad K.N., Sahoo R.C., Singh A.B. (2011). Kidney bean: A major sensitizer among legumes in asthma and rhinitis patients from India. PLoS ONE.

[57] Kumar S., Verma A.K., Sharma A., Kumar D., Tripathi A., Chaudhari B.P., Das M., Jain S.K., Dwivedi P.D. (2013). Phytohemagglutinins augment red kidney bean (*Phaseolus vulgaris* L.) induced allergic manifestations. J. Proteom..

[58] Kumar S., Sharma A., Das M., Jain S.K., Dwivedi P.D. (2014). Leucoagglutinating phytohemagglutinin: Purification, characterization, proteolytic digestion and assessment for allergenicity potential in BALB/c mice. Immunopharmacol. Immunotoxicol..

[59] He S., Zhao J., Elfalleh W., Jemaà M., Sun H., Sun X., Tang M., He Q., Wu Z., Lang F. (2018). In silico identification and in vitro analysis of B- and T-cell epitopes of the black turtle bean (*Phaseolus vulgaris* L.) lectin. Cell Physiol. Biochem..

[60] Zhao J., He S., Tang M., Sun X., Zhang Z., Ye Y., Cao X., Sun H. (2019). Low-pH induced structural changes, allergenicity and in vitro digestibility of lectin from black turtle bean (*Phaseolus vulgaris* L.). Food Chem..

[61] Lu M., Jin Y., Cerny R., Ballmer-Weber B., Goodman R.E. (2018). Combining 2-DE immunoblots and mass spectrometry to identify putative soybean (*Glycine max*) allergens. Food. Chem. Toxicol..

[62] Kleine-Tebbe J., Kagey-Sobotka A., MacGlashan D.W., Lichtenstein L.M., MacDonald S.M. (1996). Lectins do not distinguish between heterogenous IgE molecules as defined by differential activity of an IgE-dependent histamine releasing factor. J. Allergy Clin. Immunol..

[63] Risler J.L., Delorme M.O., Delacroix H., Henaut A. (1988). Amino acid substitutions in structurally related proteins. A pattern recognition approach. Determination of a new and efficient scoring matrix. J. Mol. Biol..

[64] Peumans W.J., Hao Q., Van Damme E.J.M. (2001). Ribosome-inactivating proteins from plants: More than RNA *N*-glycosidases?. FASEB J..

[65] Förster-Waldl E., Marchetti M., Schöll I., Focke M., Radauer C., Kinaciyan T., Nentwich I., Jäger S., Schmid E.R., Boltz-Nitulescu G. (2003). Type I allergy to elderberry (*Sambucus nigra*) is elicited by a 33.2 kDa allergen with significant homology to ribosomal inactivating proteins. Clin. Exp. Allergy.

[66] Szalai K., Schöll I., Förster-Waldl E., Polito A., Bolognesi E., Untermayr E., Riemer A.B., Boltz-Nitulescu G., Stirpe F., Jensen-Jarolim E. (2005). Occupational sensitization to ribosome-inactivating proteins in researchers. Clin. Exp. Allergy.

[67] Jimenez P., Cabrero P., Basterrechea J.E., Tejero J., Cordoba-Diaz D., Girbes T. (2013). Isolation and molecular characterization of two lectins from dwarf elder (*Sambucus ebulus* L.) blossoms related to the Sam n 1 allergen. Toxins.

[68] Luther P., Sehrt I., Bergmann K.C., Reutgen H. (1978). Allergy and lectins: Action between IgE-mediated histamine release and glycoproteins from *Viscum album* L. (mistletoe). Acta Biol. Med. Ger..

[69] Yoon T.J., Yoo Y.C., Kang T.B., Her E., Kim S.H., Kim K., Azuma I., Kim J.B. (2001). Cellular and humoral adjuvant activity of lectins isolated from Korean mistletoe (*Viscum album colaratum*). Int. Immunopharmacol..

[70] Reyes-López C.A., Hernández-Santoyo A., Pedraza-Escalona M., Mendoza G., Hernández-Arana A., Rodríguez-Romero A. (2004). Insights into a conformational epitope of Hev b 6.02 (hevein). Biochem. Biophys. Res. Commun..

[71] Pedraza-Escalona M., Becerril-Luján B., Agundis C., Domínguez-Ramírez L., Pereyra A., Riaño-Umbarila L., Rodríguez-Romero A. (2009). Analysis of B-cell epitopes from the allergen Hev b 6.02 revealed by using blocking antibodies. Mol. Immunol..

[72] Alenius H., Kalkkinen N., Lukka M., Reunala T., Turjanmaa K., Mäkinen-Kiljunen S., Yip E., Palosuo T. (1995). Prohevein from the rubber tree (*Hevea brasiliensis*) is a major latex allergen. Clin. Exp. Allergy.

[73] Chen Z., Posch A., Lohaus C., Raulf-Heimsoth M., Meyer H.E., Baur X. (1997). Isolation and identification of hevein as a major IgE-binding polypeptide in *Hevea* latex. J. Allergy Clin. Immunol..

[74] Karisola P., Kotovuori A., Poikonen S., Niskanen E., Kalkkinen N., Turjanmaa K., Palosuo T., Reunala T., Alenius H., Kulomaa M.S. (2005). Isolated hevein-like domains, but not 31-kD endochitinases, are responsible for IgE-mediated in vitro and in vivo reactions in latex-fruit syndrome. J. Allergy Clin. Immunol..

[75] Mikkola J.H., Alenius H., Kalkkinen N., Turjanmaa K., Palosuo T., Reunala T. (1998). Hevein-like protein domains as a possible cause for allergen cross-reactivity between latex and banana. J. Allergy Clin. Immunol..

[76] Díaz-Perales A., Collada C., Blanco C., Sánchez-Monge R., Carrillo T., Aragoncillo C., Salcedo G. (1998). Class I chitinases with hevein-like domain, but not class II enzymes, are relevant chestnut and avocado allergens. J. Allergy Clin. Immunol..

[77] Sánchez-Monge R., Blanco C., López-Torrejón G., Cumplido J., Recas M., Figueroa J., Carrillo T., Salcedo G. (2006). Differential allergen sensitization patterns in chestnut allergy with or without associated latex-fruit syndrome. J. Allergy Clin. Immunol..

[78] Chen Z., Posch A., Cremer R., Raulf-Heimsoth M., Baur X. (1998). Identification of hevein (Hev b 6.02) in *Hevea* latex as a major cross-reacting allergen with avocado fruit in patients with latex allergy. J. Allergy Clin. Immunol..

[79] Posch A., Wheeler C.H., Chen Z., Flagge A., Dunn M.J., Papenfuss F., Raulf-Heimsoth M., Baur X. (1999). Class I endochitinase containing a hevein domain is the causative allergen in latex-associated avocado allergy. Clin. Exp. Allergy.

[80] Gamboa P.M., Sánchez-Monge R., Díaz-Perales A., Salcedo G., Ansótegui J., Sanz M.L. (2005). Latex-vegetable syndrome due to custard apple and aubergine, new variations of the hevein symphony. J. Investig. Allergol. Clin. Immunol..

[81] Danhash N., Wagemakers C.A., van Kan J.A., de Wit P.J. (1993). Molecular characterization of four chitinase cDNAs obtained from *Cladosporium fulvum*-infected tomato. Plant Mol. Biol..

[82] O’Riordain G., Radauer C., Hoffmann-Sommergruber K., Adhami F., Peterbauer C.K., Blanco C., Godnic-cvar J., Scheiner O., Ebner C., Breiteneder H. (2002). Cloning and molecular characterization of the *Hevea brasiliensis* allergen Hev b 11, a class I chitinase. Clin. Exp. Allergy.

[83] Blanco C., Díaz-Perales A., Collada C., Sánchez-Monge R., Aragoncillo C., Castillo R., Ortega N., Alvarez M., Carrillo T., Salcedo G. (1999). Class I chitinases as potential panallergens involved in the latex-fruit syndrome. J. Allergy Clin. Immunol..

[84] Palosuo T., Panzani R.C., Singh A.B., Ariano R., Alenius H., Turjanmaa K. (2002). Allergen cross-reactivity between proteins of the latex from *Hevea brasiliensis*, seeds and pollen of *Ricinus communis*, and pollen of *Mercurialis annua*, members of the Euphorbiaceae family. Allergy Asthma Proc..

[85] Wagner S., Breiteneder H. (2002). The latex-fruit syndrome. Biochem. Soc. Trans..

[86] Swanson M.D., Boudreaux D.M., Salmon L., Chugh J., Winter H.C., Meagher J.L., André S., Murphy P.V., Oscarson S., Roy R. (2015). Engineering a therapeutic lectin by uncoupling mitogenicity from antiviral activity. Cell.

[87] Krithika N., Pramod S.N., Mahesh P.A., Venkatesh Y.P. (2018). Banana lectin (BanLec) induces non-specific activation of basophils and mast cells in atopic subjects. Eur. Ann. Allergy Clin. Immunol..

[88] Stojanović M.M., Zivković I.P., Petrusić V.Z., Kosec D.J., Dimitrijević L.A., Gavrović-Jankulović M.D. (2010). In vitro stimulation of Balb/c and C57 BL/6 splenocytes by a recombinantly produced banana lectin isoform results in both proliferation of T cells and an increased secretion of interferon-γ. Int. Immunopharmacol..

[89] Barbosa--Lorenzi V.C., Cecilio N.T., de Almeida Buranello P.A., Pranchevicius M.C., Goldman M.H., Pereira-da-Silva G., Roque-Barreira M.C., Jamur M.C., Oliver C. (2016). Recombinant ArtinM activates mast cells. BMC Immunol..

[90] Mondal H.A., Chakrabort D., Majumder P., Roy P., Roy A., Bhattacharya S.G., Das S. (2011). Allergenicity assessment of *Allium sativum* leaf agglutinin, a potential candidate protein for developing sap sucking insect resistant food crops. PLoS ONE.

[91] Almogren A., Shakoor Z., Adam M.H. (2013). Garlic and onion sensitization among Saudi patients screened for food allergy: A hospital based study. Afr. Health Sci..

[92] Treudler R., Reuter A., Engin A.M., Simon J.C. (2015). A case of anaphylaxis after garlic ingestion: Is alliinase the only culprit allergen?. J. Investig. Allergol. Clin. Immunol..

[93] Das A., Ghosh P., Das S. (2018). Expression of *Colocasia esculenta* tuber agglutinin in Indian mustard provides resistance against *Lipaphis erysimi* and the expressed protein is non-allergenic. Plant Cell. Rep..

[94] Schouppe D., Rougé P., Lasanajak Y., Barre A., Smith D.F., Proost P., Van Damme E.J.M. (2010). Mutational analysis of the carbohydrate binding activity of the tobacco lectin. Glycoconj. J..

[95] Van Damme E.J.M., Barre A., Rougé P., Van Leuven F., Peumans W.J. (1996). The NeuAc(α2,6)-Gal/GalNAc-binding lectin from elderberry (*Sambucus nigra*) bark, a type-2 ribosome-inactivating protein with an unusual specificity and structure. Eur. J. Biochem..

[96] Rougé P., Brunet E., Borges J.P., Jauneau A., Saggio B., Bourrier T., Fancé F., Didier A., Barre A. (2011). Proteins with cupin motif as major seed allergens. Rev. Fr. Allergol..

[97] Villanueva M.A. (2002). Elimination of artifacts on native western blots arising from endogenous lectin activity. J. Biochem. Biophys. Methods.

[98] Pramod S.N., Venkatesh Y.P., Mahesh P.A. (2007). Potato lectin activates basophils and mast cells of atopic subjects by its interaction with core chitobiose of cell-bound non-specific immunoglobulin E. Clin. Exp. Immunol..

[99] Thompson J.D., Gibson T.J., Plewniak F., Jeanmougin F., Higgins D.G. (1997). The CLUSTAL-X windows interface: Flexible strategies for multiple sequence alignment aided by quality analysis tool. Nucleic Acids Res..

[100] Banerjee R., Das K., Ravishankar R., Suguna K., Surolia A., Vijayan M. (1996). Conformation, protein-carbohydrate interactions and a novel subunit association in the refined structure of peanut lectin-lactose complex. J. Mol. Biol..

[101] Hamelryck T.W., Loris R., Bouckaert J., Dao-Thi M.H., Strecker G., Imberty A., Fernandez E., Wyns L., Etzler M. (1999). Carbohydrate binding, quaternary structure and a novel hydrophobic binding site in two legume lectin oligomers from *Dolichos biflorus*. J. Mol. Biol..

[102] Dessen A., Gupta D., Sabesan S., Brewer C.F., Sacchettini J.C. (1995). X-ray crystal structure of the soybean agglutinin cross-linked with a biantennary analog of the blood group I carbohydrate antigen. Biochemistry.

[103] Casset F., Hamelryck T., Loris R., Brisson J.R., Tellier C., Dao-Thi M.H., Wyns L., Poortmans F., Pérez S., Imberty A. (1995). NMR, molecular modeling, and crystallographic studies of lentil lectin-sucrose interaction. J. Biol. Chem..

[104] Rutenber E., Katzin B.J., Ernst S., Collins E.J., Mlsna D., Ready M.P., Robertus J.D. (1991). Crystallographic refinement of ricin to 2.5 Å. Proteins.

[105] Schwefel D., Maierhofer C., Beck J.G., Seeberger S., Diederichs K., Möller H.M., Welte W., Wittmann V. (2010). Structural basis of multivalent binding to wheat germ agglutinin. J. Am. Chem. Soc..

[106] Ramachandraiah G., Chandra N.R., Surolia A., Vijayan M. (2002). Re-refinement using reprocessed data to improve the quality of the structure: A case study involving garlic lectin. Acta Crystallogr. D Biol. Crysyallogr..

[107] Berman H.M., Westbrook J., Feng Z., Gilliland G., Bhat T.N., Weissig H., Shindyalov N., Bourne P.E. (2000). The protein data bank. Nucleic Acids Res..

[108] Krieger E., Koraimann G., Vriend G. (2002). Increasing the precision of comparative models with YASARA NOVA—A self-parametrizing force field. Proteins.

[109] Buts L., Dao-Thi M.H., Loris R., Wyns L., Etzler M., Hamelryck T. (2001). Weak protein-protein interactions in lectins: The crystal structure of a vegetative lectin from the legume *Dolichos biflorus*. J. Mol. Biol..

[110] Shetty K.N., Latha V.L., Rao R.N., Nadimpalli S.K., Suguna K. (2013). Affinity of galactose-specific legume lectin from *Dolichos lablab* to adenine revealed by X-ray crystallography. IUBMB Life.

[111] Nagae M., Soga K., Morita-Matsumoto K., Hanashima S., Ikeda A., Yamamoto K., Yamaguchi Y. (2014). Phytohemagglutinin from *Phaseolus vulgaris* (PHA-E) displays a novel glycan recognition-mode using a common legume lectin fold. Glycobiology.

[112] Nagae M., Kanagawa M., Morita-Matsumoto K., Hanashima S., Kizuka Y., Taniguchi N., Yamaguchi Y. (2016). Atomic visualization of a flipped-back conformation of bisected glycans bound to specific lectins. Sci. Rep..

[113] Laskowski R.A., MacArthur M.W., Moss D.S., Thornton J.M. (1993). PROCHECK: A program to check the stereochemistry of protein structures. J. Appl. Crystallogr..

[114] Melo F., Feytmans E. (1998). Assessing protein structures with a non-local atomic interaction energy. J. Mol. Biol..

[115] Benkert P., Biasini M., Schwede T. (2011). Toward the estimation of the absolute quality of individual protein structure models. Bioinformatics.

[116] Arnold K., Bordoli L., Kopp J., Schwede T. (2006). The SWISS-MODEL workspace: A web-based environment for protein structure homology modelling. Bioinformatics.

[117] Pettersen E.F., Goddard T.D., Huang C.C., Couch G.S., Greenblatt D.M., Meng E.C., Ferrin T.E. (2004). UCSF Chimera—A visualization system for exploratory research and analysis. J. Comput. Chem..

